# Decomposition of Iodoform Solutions

**Published:** 1890-02

**Authors:** 


Decomposition of Iodoform Solutions.--According to Carles (*Moniteur de la Pharm.*), when an ethereal solution of iodoform approaches the point of saturation it decomposes with great facility, and assumes a color resembling that of iodine tincture. A few drops of this altered solution will turn starch paper blue, showing that part of the iodine has been set free. In general, the decomposition is proportioned to the degree of saturation. When the ether is anhydrous and chemically pure, the same change takes place as with the medicinal article. By alcoholized ether decomposition is retarded, as also if the solution be exposed to the action of light. Air and ozone are without influence in this direction. The amount of iodine thus liberated is less than the phenomena would seem to indicate. It may be estimated by shaking a globule of mercury in the solution, when the free iodine will unite with the metal, forming proto-iodide of mercury, and the solution, being no longer saturated, will become more stable. Iodoform solutions thus partially decomposed may be employed in surgery without any inconvenience. Solutions of iodoform in chloroform are similarly acted on.—*Druggists' Circular.*

